# Antibody Response Against SARS-CoV-2 Spike Protein in People with HIV After COVID-19 Vaccination

**DOI:** 10.3390/vaccines13050480

**Published:** 2025-04-29

**Authors:** María José Muñoz-Gómez, Pablo Ryan, Marta Quero-Delgado, María Martin-Vicente, Guillermo Cuevas, Jorge Valencia, Eva Jiménez, Natalia Blanca-López, Samuel Manzano, Juan Ignacio Lazo, Vicente Mas, Mónica Vázquez, Daniel Sepúlveda-Crespo, Juan Torres-Macho, Isidoro Martínez, Salvador Resino

**Affiliations:** 1 Unidad de Infección Viral e Inmunidad, Centro Nacional de Microbiología, Instituto de Salud Carlos III, Majadahonda, 28222 Madrid, Spain; mjose.munoz@isciii.es (M.J.M.-G.); marta.quero@isciii.es (M.Q.-D.); maria.mvicente92@gmail.com (M.M.-V.); daniel.sepulveda@isciii.es (D.S.-C.); 2 Centro de Investigación Biomédica en Red en Enfermedades Infecciosas (CIBERINFEC), Instituto de Salud Carlos III, 28029 Madrid, Spain; pablo.ryan@salud.madrid.org; 3 Servicio de Medicina Interna, Hospital Universitario Infanta Leonor, 28031 Madrid, Spain; guillermo.cuevas@salud.madrid.org (G.C.); jorge.valencia@salud.madrid.org (J.V.); natalia.blanca@salud.madrid.org (N.B.-L.); samuel.manzano@salud.madrid.org (S.M.); juan.torresm@salud.madrid.org (J.T.-M.); 4 Departamento de Medicina, Facultad de Medicina, Universidad Complutense de Madrid, 28040 Madrid, Spain; 5 Instituto de Investigaciones Sanitarias Gregorio Marañón (IiSGM), 28009 Madrid, Spain; 6 Servicio de Medicina Preventiva, Hospital Universitario Infanta Leonor, 28031 Madrid, Spain; 7 Servicio de Urgencias, Hospital Universitario Infanta Leonor, 28031 Madrid, Spain; juanignacio.lazo@salud.madrid.org; 8 Unidad de Biología Viral, Centro Nacional de Microbiología, Instituto de Salud Carlos III, 28222 Madrid, Spain; vmas@isciii.es (V.M.); mvazquez@isciii.es (M.V.)

**Keywords:** HIV, SARS-CoV-2, spike glycoprotein, antibody, vaccine

## Abstract

**Background/Objectives:** People with HIV (PWH) often have a suboptimal response to vaccines, raising concerns regarding the efficacy of coronavirus disease 2019 (COVID-19) vaccines in this population. We aimed to evaluate the humoral immune response to the B.1 lineage and Omicron variant in PWH on antiretroviral therapy (ART) following COVID-19 vaccination. **Methods**: We conducted a prospective study of 19 PWH on ART who received a two-dose series of the COVID-19 mRNA vaccine and a booster six months later. Participants without HIV infection (n = 25) were included as a healthy control (HC) group. The humoral response to the COVID-19 vaccine (anti-SARS-CoV-2 S IgG levels and ability to block ACE2-S interaction) against both the original B.1 lineage and the Omicron variant was assessed using immunoassays. **Results**: The humoral response in PWH was very strong (geometric mean fold rise, GMFR > 8) after the second dose and strong (GMFR > 4) after the booster dose for both the B.1 lineage and the Omicron variant. We found comparable humoral responses to the B.1 lineage and Omicron variant between PWH and HC groups after the second and booster doses (q-value > 0.05). The COVID-19 vaccine generated a significantly weaker humoral response against the Omicron variant compared to the B.1 lineage in both groups (q-value < 0.05). However, this response improved after the booster dose, although it remained weaker in PWH. **Conclusions**: PWH showed a strong humoral response to the COVID-19 vaccine against B.1 and Omicron, though the Omicron response was weaker than B.1. Booster doses in PWH improved the Omicron response, but it stayed lower than B.1. Findings confirm vaccine effectiveness in PWH, stressing the critical role of boosters and potential need for updated vaccines for variants like Omicron.

## 1. Introduction

Human immunodeficiency virus (HIV) infection causes significant alterations in the immune system, particularly among people with HIV (PWH) who are not receiving antiretroviral therapy (ART). However, even in PWH who have achieved HIV suppression with ART, chronic immune impairment may persist [1], potentially contributing to suboptimal antibody production following vaccination, resulting in diminished vaccine efficacy [2]. This immune dysfunction is often characterized by incomplete immune recovery, persistent systemic inflammation, and sustained immune dysfunction. The presence of concomitant infections and/or comorbid conditions in PWH further exacerbates these conditions. Consequently, the persistent immunopathology associated with chronic HIV infection may compromise the immune response to SARS-CoV-2 and can interfere with optimal vaccine-induced immunity [3].

The clinical impact of COVID-19 among PWH on ART has shown variable outcomes. While some studies have not identified HIV infection as a significant risk factor for COVID-19-related mortality, other reports have demonstrated that PWH on ART exhibits a significantly increased risk of death compared to HIV-negative individuals [3,4,5]. As a result, PWH were designated as a priority group for COVID-19 vaccination [2]. The most effective COVID-19 vaccines in preventing severe COVID-19 are the COVID-19 mRNA vaccines, BNT162b2 (Pfizer-BioNTech) and mRNA-1273 (Moderna). In contrast, previous studies have reported reduced efficacy of the NVX-CoV2373 vaccine, a recombinant spike protein nanoparticle formulated with the Matrix-M adjuvant, in PWH compared to HIV-negative individuals [6]. However, considering that PWH may exhibit suboptimal responses to other vaccines [7], concerns remain about the effectiveness of COVID-19 mRNA vaccines in this population. Additionally, PWH were not included in clinical trials [2], so most of the available information comes from observational studies.

COVID-19 mRNA vaccines induce specific neutralizing antibodies against the spike glycoprotein (S) of Severe Acute Respiratory Syndrome Coronavirus 2 (SARS-CoV-2), which mediates the binding to the angiotensin-converting enzyme 2 (ACE2) receptor on host cells [8,9]. While numerous studies have established the safety of COVID-19 mRNA vaccines among PWH, there are conflicting data regarding the vaccine-induced humoral immune response in this population [10]. Some studies suggest that there is no difference between PWH and HIV-negative individuals concerning antibody kinetics, peak titers, and neutralization activity [11]. In contrast, other researchers have demonstrated that the vaccine-antibody response in PWH is correlated with their CD4^+^ T-cell count levels and viremia, with a compromised immune response in PWH with less than 200 CD4^+^ T-cells per mm^3^ [12,13].

SARS-CoV-2 mutates quickly, evolving into new variants that increase transmissibility and virulence or reduce vaccine efficacy [2]. Moreover, PWH, particularly those with unsuppressed HIV infection, are at a higher risk of prolonged SARS-CoV-2 viral shedding. This presents a threat not only to the individual but also to public health, as persistent infection can lead to the rapid accumulation of viral mutations, potentially contributing to the emergence of variants of concern (VOCs) [14]. The Omicron variant is highly transmissible and resistant to vaccine-induced immunity. Antibody and neutralizing responses against Omicron are significantly lower compared to those against the wild-type SARS-CoV-2 [15,16]. In the general population, completing a primary vaccination series with two doses offers limited protection against severe Omicron outcomes, but a booster dose increases protection. However, data on PWH are limited, and clear gaps persist, particularly regarding the magnitude and duration of their humoral defense against the Omicron variant. Furthermore, the degree to which lower CD4^+^ T-cell counts may compound this vulnerability requires more focused evaluation.

### Objective

We aimed to evaluate the humoral immune response against the B.1 lineage and the Omicron variant of SARS-CoV-2 induced via COVID-19 vaccination in PWH.

## 2. Materials and Methods

### 2.1. Study Design

We conducted a prospective study with 19 PWH who received the COVID-19 mRNA vaccine, based on the Wuhan-Hu-1 strain, for the first time between February 2021 and July 2021 at the Hospital Universitario Infanta Leonor (HUIL) in Madrid, Spain. We included a group of 25 healthy controls (HC) matched by age and sex who were recruited from hospital staff.

The vaccination schedule followed international guidelines [17], consisting of two doses of a COVID-19 mRNA vaccine with a 28-day interval, followed by a third booster dose six months later. Of the 44 individuals vaccinated with the first and second dose, 18 PWH and 25 HC were administered the Moderna mRNA-1273 vaccine, while one PWH received the Pfizer BNT162b2 mRNA vaccine. At the booster dose (n = 27), 15 PWH and 5 HC received the Moderna mRNA-1273 vaccine, and 7 HC received the Pfizer BNT162b2 mRNA vaccine.

The study protocol (Ref. 030-21) was approved by the HUIL Ethics Committee and conducted in accordance with the Declaration of Helsinki. All participants provided written informed consent prior to enrollment.

### 2.2. Clinical Data and Samples

Participant characteristics were collected from the hospital’s electronic medical records. Data were stored using the Research Electronic Data Capture system version 8.8.1 (REDCap, Vanderbilt University, Nashville, TN, USA) [18].

Blood samples were drawn at three points: at the first COVID-19 vaccination (baseline), around four weeks after the second vaccine dose, and approximately ten weeks after the booster dose. Plasma was isolated from blood samples collected in ethylene-diamine-tetraacetic acid tubes using a Ficoll gradient and frozen at −80 °C for later analysis. Prior to analysis, plasma samples were heat-inactivated at 56 °C for 30 min to ensure viral inactivation.

### 2.3. Previous SARS-CoV-2 Infection

Participant plasma samples were tested for SARS-CoV-2 infection at the baseline, after the second dose, and after the booster dose of the COVID-19 vaccine. The test detected IgG, IgA, and IgM antibodies against SARS-CoV-2 N protein using a commercial enzyme-linked immunosorbent assay (ELISA) (Platelia SARS-CoV-2 Total Ab, Bio-Rad Laboratories Inc., Hercules, CA, USA). A sample was classified as positive if the optical density ratio between the test and control samples was greater than or equal to 1 (ratio ≥ 1.0). This cutoff has a sensitivity of 94.7% and a specificity of 97.5% when used two weeks after the RT-qPCR positive detection [19].

### 2.4. Immunoassay for Anti-SARS-CoV-2 S IgG Quantification

The pαH plasmid encoding the ectodomain (residues 1–1208) of the SARS-CoV-2 spike (S) protein, derived from the 2019-nCoV reference sequence (GenBank: MN908947) and stabilized in its prefusion conformation, was generously supplied by Dr. Jason McLellan (University of Texas at Austin, USA). Site-directed mutagenesis was employed to generate two HexaPro constructs designed to enhance the yield of the stabilized prefusion form of the S protein. For the first construct (B.1 lineage), the following substitutions were made: D614G (aspartic acid to glycine at position 614), a “GSAS” substitution replacing the furin cleavage site at residues 682–685, and six proline substitutions at positions 817, 892, 899, 942, 986, and 987 to promote conformational stability. For the second construct (SARS-CoV-2 S Omicron, BA.1 sublineage), the natural cleavage site “RRAR” (residues 682–685) was included, along with these Omicron-specific mutations: A67V, Δ69–70, T95I, G142D/Δ143–145, Δ211/L212I, ins214EPE, G339D, S371L, S373P, S375F, K417N, N440K, G446S, S477N, T478K, E484A, Q493R, G496S, Q498R, N501Y, Y505H, T547K, D614G, H655Y, N679K, P681H, N764K, D796Y, N856K, Q954H, N969K, and L981F. For trimer formation and downstream purification, the C-terminal region of the S ectodomain was engineered to include the T4 fibritin foldon trimerization domain, an HRV3C protease cleavage site, and an 8 × His affinity tag. Additionally, a version of the S protein with a locked closed conformation incapable of ACE2 binding was generated by introducing cysteine residues at positions S383C and D985C into the HexaPro construct. A separate plasmid was constructed encoding the residues 1–165 of ACE2, the cell receptor for the SARS-CoV-2 S protein, fused to a StrepTag for purification purposes.

Transient transfection of FreeStyle 293F cells (Life Technologies, Carlsbad, CA, USA) was performed using the plasmid encoding the S ectodomain. The recombinant S protein was harvested from the clarified culture supernatant and purified using Ni-NTA affinity chromatography (Cytiva, Uppsala, Sweden). After washing with 20 mM Na_2_HPO_4_ pH 7.4, 200 mM NaCl, and 20 mM imidazole, bound proteins were eluted through a gradient increase in imidazole concentration, reaching up to 300 mM in the same buffer system. Eluted fractions were concentrated using Amicon centrifugal filters (Millipore, Burlington, MA, USA) and buffer-exchanged into 20 mM Na_2_HPO_4_ pH 7.4, 200 mM NaCl to remove imidazole prior to size exclusion chromatography on a Superose 6 10/300 GL column (Cytiva, Uppsala, Sweden), equilibrated in the same buffer. Protein purity and structural integrity were evaluated using SDS-PAGE, followed by Coomassie blue staining under reducing conditions. The ACE2-StrepTag fusion protein was expressed under similar conditions and purified using the Strep-Tag affinity system, followed by further polishing on a Superdex 200 column (Cytiva, Uppsala, Sweden).

Antibodies against the S protein were titrated using an ELISA assay. Serial 1:3 dilutions of plasma samples (starting from 1:50 and ending at 1:36, 450) were incubated with the purified S protein ectodomain. Ninety-six-well plates were pre-coated with 200 ng of the recombinant S protein per well. On the following day, serum dilutions were applied, and antigen-antibody binding was detected through sequential incubations with horseradish peroxidase (HRP)-conjugated secondary antibody specific for human IgG (Jackson ImmunoResearch, West Grove, PA, USA). Signal development was achieved using an o-phenylenediamine dihydrochloride (OPD) substrate (Sigma-Aldrich, St. Louis, MO, USA), and absorbance was measured at 493 nm. To ensure assay reproducibility, all ELISA tests were performed in duplicate, and positive control samples were included on each plate to confirm assay consistency. One-phase exponential decay least-squares fit curves were used, and the area under the curve was calculated using GraphPad Prism 10.0 (GraphPad Software, Inc., San Diego, CA, USA).

The antibody inhibition of the ACE2-S protein interaction was tested via ELISA. Fifty nanograms per well of the S protein ectodomain were captured via a monoclonal anti-Foldon antibody, which had been pre-coated onto 96-well plates. Next, serum samples were serially diluted (1:2), starting at 1:10 and extending to 1:320). Then, one µg of the cell receptor ACE2 complexed with StrepTactin-peroxidase (Bio-Rad, Halle, Germany) was added to each well and revealed with the OPD substrate. Optical density was then measured at 493 nm. A pooled serum sample from 2016, collected from individuals who tested negative for anti-S antibodies, was used as a control. After background subtraction, the inhibition percentage for each dilution was calculated as [1 − (OD_493_ test plasma/OD_493_ control plasma)] × 100%. Using these percentages, curves were fitted to a one-phase exponential decay model using least-squares regression. The area under the curve (AUC) was then calculated using GraphPad Prism 10.0.

### 2.5. Statistical Analysis

IBM SPSS Statistics 25.0 (IBM Corp., Armonk, NY, USA) and Stata 18.0 (StataCorp, College Station, TX, USA) were used for statistical analysis. GraphPad Prism 10.0 was used to create figures. The significance level was set to q < 0.05.

The two main factors analyzed were the study population (PWH vs. HC) and the immune response to SARS-CoV-2 variants (B.1 vs. Omicron). The primary outcome was the humoral response to the COVID-19 vaccine, measured by anti-SARS-CoV-2 S IgG levels and their ability to block the ACE2-S interaction against both the original B.1 lineage and the Omicron variant after the second dose and booster dose. Humoral response data were log_10_-transformed.

Generalized linear mixed models (GLMMs) were used to calculate the geometric mean fold rise (GMFR) and its 95% CI between post- and pre-vaccination. For this study, we classified GMFR values into three categories: >8-fold rise (very strong response), 4–8-fold rise (strong response), and <4-fold rise (weak response).

These cutoffs are commonly adopted in the vaccine immunogenicity literature to provide straightforward interpretation thresholds, although no single definitive standard exists [20,21,22,23,24]. We selected these ranges based on prior reports using similar approaches to fold-rise categorization, as well as our group’s internal criteria, which were refined to fit our data distribution and study objectives.

Additionally, GLMMs were used to evaluate the impact of two main factors (HIV infection and SARS-CoV-2 variants) on the COVID-19 vaccine-induced humoral response, providing differences among groups. These analyses were adjusted for clinical variables (type of vaccine and previous SARS-CoV-2 infection), which were selected through a stepwise forward selection method (pin < 0.05 and pout < 0.10). *p*-values were adjusted for the false discovery rate (q-values) using the Benjamini–Hochberg procedure. The rate of non-responders (AUC = 0) was compared using the Chi-squared test.

## 3. Results

### 3.1. Patient Characteristics

The baseline characteristics of the 19 PWH included in this study are shown in Table 1. All participants were on ART, although two had a detectable viral load (>50 HIV RNA copies/mL). Additionally, six participants had a CD4 count below 200 cells/mm^3^.

### 3.2. Humoral Response to the COVID-19 Vaccine

The GMFR values of the humoral response (anti-SARS-CoV-2 S IgG and inhibition of ACE2-S interaction) were higher than 8 after the second dose and higher than 4 after the booster dose for both the B.1 lineage and the Omicron variant (Table 2). Additionally, there was generally a strong correlation between anti-SARS-CoV-2 S IgG titers and ACE2-S interaction inhibition in both the HC and PWH groups (Supplementary Figure S1).

#### 3.2.1. Humoral Response to COVID-19 Vaccine Between Study Groups

**Figure 1:**
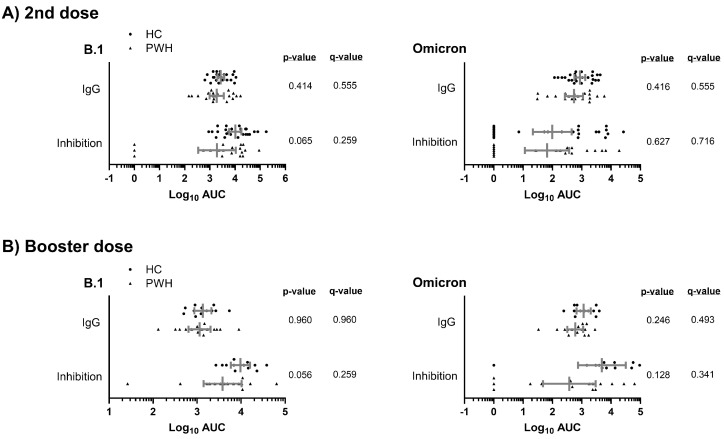
Comparison of the humoral response to COVID-19 vaccine (anti-SARS-CoV-2 S IgG and inhibition of ACE2-S interaction) between study groups against the B.1 lineage and the Omicron variant after the second (**A**) and a booster dose (**B**) of the COVID-19 vaccine. Statistics: The graph shows the geometric means and 95% confidence intervals in gray lines. *p*-values were calculated using generalized linear mixed models. Abbreviations: HC, healthy controls; PWH, people with HIV; Log_10_, base-10 logarithm; AUC, area under the curve; IgG, immunoglobulin G.

We found similar humoral responses (anti-SARS-CoV-2 S IgG and inhibition of ACE2-S interaction) to the B.1 lineage and Omicron variant between PWH and HC groups after the second and booster doses of the COVID-19 vaccine (q-value > 0.05; Figure 1A,B). Therefore, these results indicate that the humoral response in the PWH group may be comparable to that of the HC group.

#### 3.2.2. Humoral Response to COVID-19 Vaccine Between SARS-CoV-2 Variants

**Figure 2:**
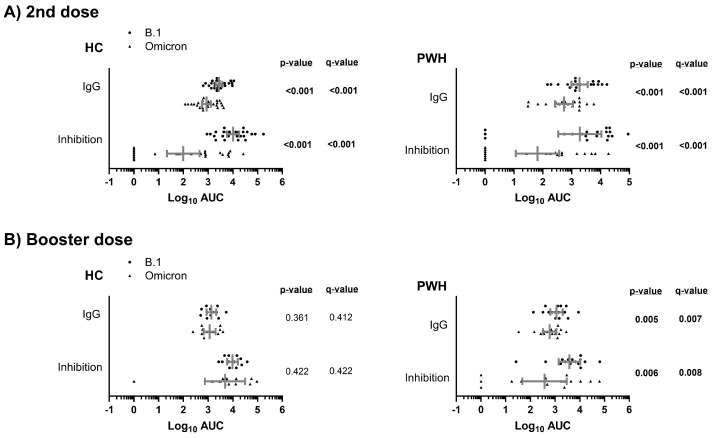
Comparison of the humoral response to COVID-19 vaccine (anti-SARS-CoV-2 S IgG and inhibition of ACE2-S interaction) between SARS-CoV-2 variants in study groups after the second (**A**) and booster dose (**B**) of the COVID-19 vaccine. Statistics: The graph shows the geometric means and 95% confidence intervals in gray lines. *p*-values were calculated using generalized linear mixed models. Significant differences are shown in bold. Abbreviations: HC, healthy controls; PWH, people with HIV; Log_10_, base-10 logarithm; AUC, area under the curve; IgG, immunoglobulin G.

After the second dose, both PWH and HC groups exhibited significantly weaker humoral responses to the Omicron variant compared to the B.1 lineage (q-value < 0.05; Figure 2A). However, following the booster dose, this significant difference in humoral responses between the B.1 lineage and the Omicron variant was only observed within the PWH group (q-value < 0.05; Figure 2B). Nevertheless, the differences in the rate of non-responders among PWH for ACE2-S interaction inhibition (AUC = 0) against B.1 and Omicron were not significant after the booster dose (Supplementary Figure S2).

Therefore, the COVID-19 vaccine generated a significantly weaker response (measured by both IgG levels and inhibition capacity) against the Omicron variant compared to the B.1 lineage in both the HC and PWH groups after the second vaccine dose. However, this response improved after the booster dose only in the HC group, while it remained statistically significantly weaker in the PWH group.

## 4. Discussion

Although PWH sometimes exhibits weaker vaccine responses, particularly at lower CD4^+^ counts, our findings indicate that mRNA-based COVID-19 vaccination remains broadly effective. This study directly compared humoral responses to the B.1 lineage and Omicron variant in PWH and HIV-negative controls. The key observations were as follows: (1) PWH mounted strong humoral responses following both the second and booster vaccine doses. (2) The difference in humoral response to the B.1 lineage and the Omicron variant, after both the second and booster doses, was not statistically significant when the PWH and HC groups were compared (q-value ≥ 0.05). (3) In the PWH and HC groups, the humoral response to the Omicron variant was weaker than the response to the B.1 lineage. However, this response improved after the booster dose in the HC group, while it remained weaker in the PWH group.

Our data confirm a robust humoral response to the COVID-19 vaccine in PWH. The results demonstrate a substantial increase in antibody levels against the B.1 lineage and the Omicron variant. The GMFR values were ≥8 after the second dose and ≥4 after the booster dose, corresponding to a “very strong” and “strong” immune response, respectively. These results align with previous studies [25], which indicate that consecutive vaccine doses enhance the immune response in PWH, ultimately helping protect this population from SARS-CoV-2 infection. Our data underscore the potential benefits of COVID-19 vaccination in protecting PWH against SARS-CoV-2 infection.

Notably, we found no significant differences in antibody levels and ACE2–S interaction inhibition between PWH and HC, consistent with other mRNA vaccine studies reporting similar immune profiles in these populations [26,27,28,29]. Notably, in these studies, the PWH were on ART with suppressed HIV viral loads and exhibited higher baseline CD4 counts compared to studies reporting diminished IgG responses in PWH relative to the HC group [28,30]. In our study, patients were on suppressive ART with a median lower than 300 CD4^+^ T-cells/mm^3^, and 31.6% had lower than 200 CD4^+^ T-cells/mm^3^. Nevertheless, even under these conditions, patients on suppressive ART mounted a robust response.

After the second dose, Omicron-specific IgG levels and ACE2–S interaction inhibition were notably lower than those targeting the B.1 lineage, consistent with Omicron’s multiple spike protein mutations that confer partial immune evasion [31]. However, the response to Omicron significantly improved in the HC group following the administration of a booster dose, but not among PWH. Consistent with these observations, previous studies have shown that, despite the immune evasion of the Omicron variant to COVID-19 vaccines, booster doses are critical to enhancing the efficacy of the immune response [32,33]. However, PWH maintained a weaker humoral response to Omicron compared to the B.1 lineage following the booster dose, indicating a higher risk of Omicron infection, as previously published data suggest [3,34]. Thus, our results underscore the crucial role of booster doses in enhancing protection against Omicron, despite suboptimal humoral immunity. In this context, implementing immune monitoring to guide individualized booster schedules—particularly in individuals with low CD4^+^ T-cell counts—may enhance the immunogenicity and overall effectiveness of vaccination strategies in PWH.

Beyond the immunological findings, the real-world clinical impact is paramount. While our study demonstrates that PWH can generate robust antibody responses that are not statistically different from those of HIV-negative individuals, it is crucial to ascertain whether these responses confer actual protection against clinical outcomes. Although several studies have demonstrated that the humoral response to SARS-CoV-2 declines significantly over time, data remain limited regarding PWH, who may experience a more rapid decline in protection and breakthrough infections [3]. In this context, previous studies have reported comparable humoral and cellular immune responses between PWH and HIV-negative controls up to 11 months following a booster dose of the BNT162b2 vaccine. However, longer-term studies are warranted, particularly those evaluating immune responses to VOCs, such as Omicron [29]. Future research should prospectively monitor breakthrough infections and disease severity alongside the persistence of both humoral and cellular immune responses following COVID-19 vaccination, including extended follow-up periods after booster administration. It will be particularly important to incorporate analyses of cellular immunity, such as T-cell responses, to understand the protective immune landscape better, especially among individuals with low CD4^+^ T-cell counts. While this study focused on humoral responses, growing evidence indicates that cellular immunity may play a pivotal role in long-term protection, especially in immunocompromised populations. Previous research has shown that robust T-cell responses can compensate for waning antibody levels and provide cross-variant protection [35,36]. Including assessments such as IFN-γ ELISpot or flow cytometry-based intracellular cytokine staining in future work would provide a more comprehensive immune profile in PWH. Furthermore, investigations should explore the potential influence of co-infections, chronic immune activation, and unsuppressed HIV replication on the magnitude and durability of vaccine-induced immunity in this population. This approach will provide a comprehensive understanding of how humoral and cellular immunity collectively contribute to SARS-CoV-2 protection in PWH. By combining clinical follow-up with detailed immunological assessments, we can determine how factors like baseline CD4^+^ levels, viral suppression duration, and booster intervals optimize vaccine strategies and improve long-term outcomes for this population.

### Study Limitations

This study involved several limitations that should be considered when interpreting the results. First, the sample size was limited, which reduces statistical power and potentially increases the risk of both type I and type II errors. Second, the prospective nature of the study may have introduced potential attrition bias, as some participants did not complete all follow-up visits. Third, the sample size was insufficient to perform detailed subgroup analyses (e.g., examining individuals with CD4^+^ < 200 cells/mm^3^ or those who developed hybrid immunity through prior infection). Finally, our findings are primarily relevant to PWH who are on suppressive ART and may not apply to PWH who are not on ART with severe immune deficiency. Continued investigation is needed to track responses to emerging SARS-CoV-2 variants and optimize vaccination strategies for these vulnerable groups.

## 5. Conclusions

PWH on suppressive ART demonstrated a strong humoral response to the COVID-19 vaccine against both the B.1 lineage and the Omicron variant. However, the humoral response to the Omicron variant was weaker than that to the B.1 lineage, and although boosters improved this response, it remained comparatively lower than the B.1 response within the PWH. These findings affirm vaccine effectiveness in PWH but underscore the critical role of boosters and the potential need for updated vaccines to address evolving variants like Omicron.

## Figures and Tables

**Table 1:** Characteristics of people with HIV infection at baseline.

Characteristics	Data
No.	19
Age (years)	41 (33; 50)
Sex (male)	15 (78.9%)
Previous SARS-CoV-2 Infection (+)	6 (31.6%)
HIV infection	
ART	19 (100.0%)
CD4^+^ count (cells/mm^3^)	232 (158; 278)
CD4^+^ count < 200 cells/mm^3^	6 (31.6%)
HIV viral load > 50 copies/mL	2 (10.5%)

Statistics: Values are expressed as the median (Q1; Q3) and absolute count (percentage). Abbreviations: HIV, human immunodeficiency virus; SARS-CoV-2, severe acute respiratory syndrome coronavirus 2; ART, antiretroviral treatment.

**Table 2:** Humoral response in PWH generated via COVID-19 vaccine stratified by SARS-CoV-2 variants.

	Baseline	After the Second Dose		After the Booster Dose	
	GMT (95% CI)	GMT (95% CI)	GMFR (95% CI)	GMT (95% CI)	GMFR (95% CI)
**Wuhan (B.1)**					
IgG antibody titers	0.7 (0.2; 2.6)	1866.5 (975.4; 3571.7)	30.4 (18.7; 49.6)	1134.9 (638.1; 2018.4)	5 (3.5; 7.1)
Inhibition ACE2-S titer	0.3 (0.1; 1.5)	1326.5 (159.6; 11,027.6)	35.1 (14.7; 83.9)	3780.1 (1395.5; 10,239.6)	7.6 (5.5; 10.5)
**Omicron**					
IgG antibody titers	0.3 (0.1; 0.8)	539.5 (262.3; 1109.6)	26 (18.4; 36.8)	597.3 (319.3; 1117.4)	5.3 (4.1; 6.7)
Inhibition ACE2-S titer	0.1 (0.1; 0.1)	28 (3; 264.8)	11.6 (5.2; 25.9)	234.3 (18.9; 2906.4)	5.4 (3.7; 8)

Statistics: Values are expressed as a geometric mean fold rise (95% confidence interval). Data were calculated using adjusted generalized linear mixed models (GLMMs; see Section 2.5). Abbreviations: Values are expressed as a geometric mean fold rise (95% confidence interval). Data were calculated using adjusted generalized linear mixed models (GLMMs; see Section 2.5).

## Data Availability

The datasets used and/or analyzed during the current study are available from the corresponding author upon reasonable request.

## Supplementary Materials

The following supporting information can be downloaded at https://www.mdpi.com/article/10.3390/vaccines13050480/s1, Figure S1: Correlation between plasma IgG antibody levels against SARS-CoV-2 S protein (B.1 lineage and Omicron variant) and its capacity to inhibit the ACE2 receptor-S protein interaction in healthy controls and people with HIV after the second dose (**A**) and booster dose (**B**); Figure S2: Rates of PWH non-responders in the inhibition of the ACE2-S interaction (AUC = 0), according to SARS-CoV-2 variants, after the second dose and the booster dose of the COVID-19 vaccine.

## Abbreviations

The following abbreviations are used in this manuscript:

SARS-CoV-2	Severe acute respiratory syndrome coronavirus 2
COVID-19	Coronavirus disease 2019
PWH	People with human immunodeficiency virus
ART	Antiretroviral therapy
S	Spike glycoprotein
ACE2	Angiotensin-converting enzyme 2
HUIL	Hospital Universitario Infanta Leonor
HC	Healthy controls
IgG	Immunoglobulin G
IgM	Immunoglobulin M
IgA	Immunoglobulin A
ELISA	Enzyme-linked immunosorbent assay
Anti-SARS-CoV-2 S IgG	IgG against the SARS-CoV-2 spike protein
AUC	Area under the curve
GLMM	Generalized linear mixed models
GMFR	Geometric mean fold rise
95%CI	95% confidence interval
GMT	Geometric mean titer
