# Supplementary material for: Antibody Response Against SARS-CoV-2 Spike Protein in People with HIV After COVID-19 Vaccination
Source: Vaccines (Basel). 2025 Apr 29;13(5):480. doi: 10.3390/vaccines13050480 (PMC12115449; doi:10.3390/vaccines13050480)

**Supplementary Figure S1.** Correlation between plasma IgG antibody levels against SARS-CoV-2 S protein (B.1 lineage and Omicron variant) and its capacity to inhibit the ACE2 receptor-S protein interaction in healthy controls and people with HIV after the second dose (A) and booster dose (B).

**Statistics**: Correlation analysis was performed using the Spearman test.

**Abbreviations**: AUC, the area under the curve; IgG, anti-SARS-CoV-2 S IgG; ACE2, angiotensin-converting enzyme 2; HC, healthy controls; PWH, people with HIV.

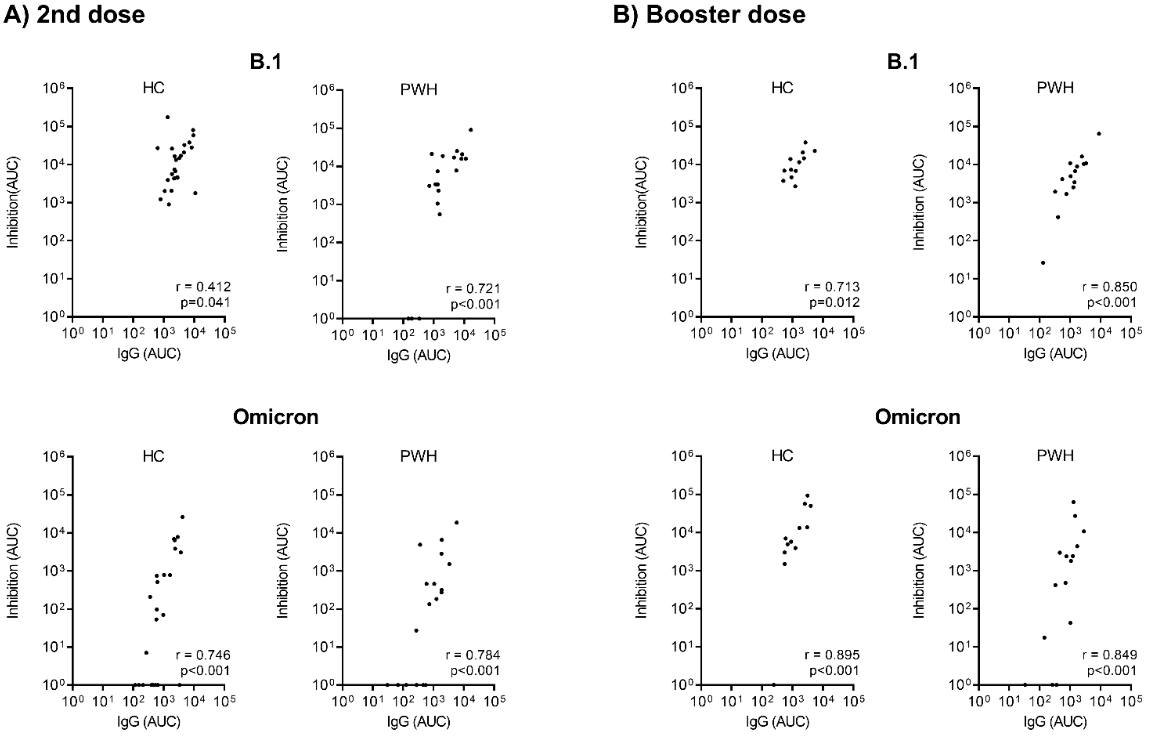


**Supplementary Figure S2**. Rates of PWH non-responders in the inhibition of the ACE2-S interaction (AUC = 0), according to SARS-CoV-2 variants, after the second dose and the booster dose of the COVID-19 vaccine.

**Statistics**: P-values were calculated using the Chi-square test.

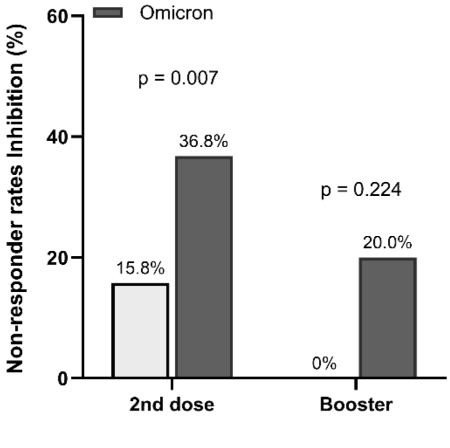

Supplement: Supplementary file 1 [file vaccines-13-00480-s001.zip › vaccines-3566128-supplementary.pdf]
